# Supplementary material for: Complex‐centric proteome profiling by SEC‐SWATH‐MS
Source: Mol Syst Biol. 2019 Jan 14;15(1):e8438. doi: 10.15252/msb.20188438 (PMC6346213; doi:10.15252/msb.20188438)
Supplement: Supplementary file 7 — Dataset EV6 [file MSB-15-e8438-s007.zip › feature_plots_bioplex/Q8WWY6.pdf]

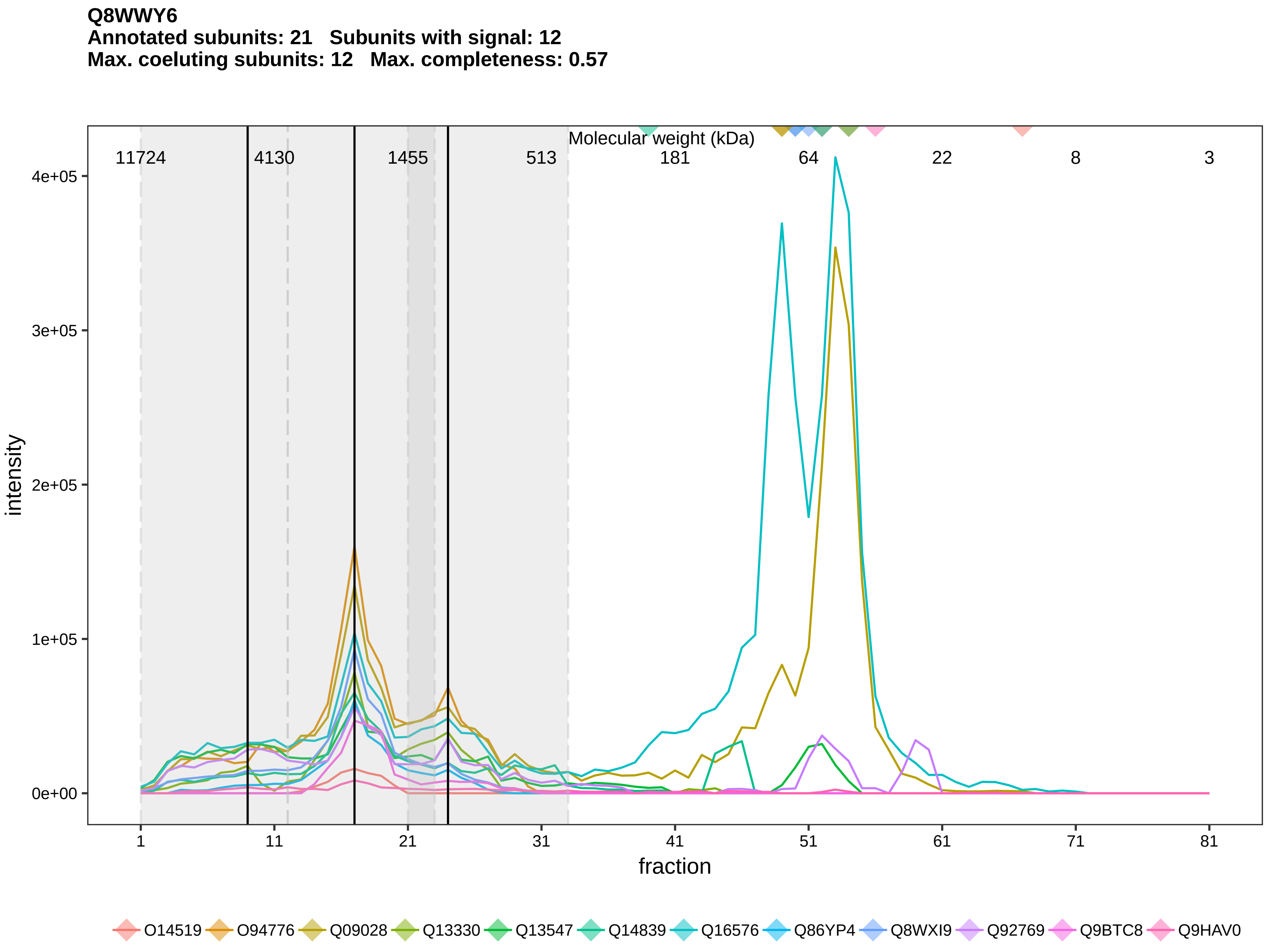
Q8WWY6
Annotated subunits: 21 Subunits with signal: 12
Max. coeluting subunits: 12 Max. completeness: 0.57
Molecular weight (kDa)
11724
4130
1455
513
181
64
22
8
3
intensity
4e+05
3e+05
2e+05
1e+05
0e+00
1
11
21
31
41
51
61
71
81
fraction
O14519
O94776
Q09028
Q13330
Q13547
Q14839
Q16576
Q86YP4
Q8WXI9
Q92769
Q9BTC8
Q9HAV0
